# Editorial: Clinical experience of open cerebral revascularization (bypass surgery) for the management of ischemic or hemorrhagic stroke

**DOI:** 10.3389/fneur.2023.1354100

**Published:** 2024-01-04

**Authors:** Alexander F. Kuffer, Danielle Golub, Amir R. Dehdashti

**Affiliations:** Northwell Health Neurosurgery, North Shore University Hospital, Manhasset, NY, United States

**Keywords:** cerebral bypass surgery, clinical experience, Moyamoya, complex aneurysm, editorial, STA-MCA bypass surgery

The fundamental concept of cerebral revascularization involves redirecting blood flow through a conduit from a robust inflow region to an undersupplied area of the brain. Indications for this procedure encompass flow augmentation for ischemia or flow preservation in complex aneurysm or tumor surgeries.

The journey of clinical experience in cerebral bypass surgery commenced in 1967 with Yasargil's pioneering superficial temporal artery (STA) to middle cerebral artery (MCA) bypass (1). This milestone was not just a testament to surgical skill but also to advancements such as the introduction of the operative microscope, development of microinstruments, bipolar forceps for vessel coagulation, and progress in neuroanesthesiology. The late 1970s witnessed the widespread clinical application of cerebral revascularization, inspired by the success of coronary bypass surgery.

Despite these advancements, the 1985 EC/IC study (2) failed to confirm the efficacy of extracranial-intracranial bypass surgery in preventing ischemic strokes for patients with symptomatic atherosclerotic disease of the internal carotid artery. While technically successful, the clinical outcomes did not demonstrate an advantage for surgery. Criticisms were directed at the study's lack of differentiation between hemodynamic and thromboembolic causes of stroke, as well as the absence of standardized surgical procedures across study sites. The initial enthusiasm for cerebral bypass surgery waned, only to be rekindled by subsequent diagnostic technology developments.

The preoperative evaluation of the oxygen extraction fraction (OEF) using positron emission tomography (PET) emerged as a pivotal step for precise patient selection. Elevated OEF or abnormal responses to acetazolamide challenge identified patients at a higher stroke risk, making them promising candidates for cerebral revascularization. The Japanese EC-IC Bypass Trial (3) (JET) demonstrated a lower stroke recurrence in the bypass group, but the Carotid Occlusion Surgery Study (4) (COSS) was halted due to a high 30-day event rate and lack of significant outcome benefits in the surgery group.

Post-COSS, bypass surgery receded from the standard armamentarium against atherosclerotic vascular disease. It became confined to specialized high-volume centers and applied only to specific patient populations, such as those with repeated strokes or hemodynamic symptoms despite optimal medical and endovascular treatment, and acute stroke patients with small strokes and extended penumbra, harboring considerable brain tissue at risk. Several studies thereafter showed benefit for cerebral bypass in well selected patients (5).

In the context of flow augmentation for Moyamoya vasculopathy, the outcomes are favorable, revealing reductions in ischemic and hemorrhagic strokes and protection against cognitive decline (6). The Japanese Adult Moyamoya (JAM) trial (7) in 2014 showcased a significant preventive effect of bypass surgery against rebleeding. Zhang et al., in this Research Topic, present a single-center case series supported by a systematic literature review, demonstrating a substantial reduction in rebleeding, ischemic events, and mortality for patients with hemorrhagic Moyamoya disease in East Asia. Lu et al. introduce refinements in the bypass technique for Moyamoya disease, emphasizing a modified approach that separates both branches of the STA and selectively performs bypasses with M4 branches, providing improved blood flow to multiple ischemic areas while reducing hyperperfusion and maintaining scalp blood supply.

Another unequivocal indication for EC-IC bypass surgery is flow preservation in surgically or endovascularly untreatable aneurysms. Chen et al., through a systematic review, involving 21 studies and 915 patients, affirm the procedure's high safety profile. Wang et al. shed light on the management of complex intracranial aneurysms with the in situ side-to-side strategy, particularly effective when vital artery sacrifice is unavoidable. Wang and Tong propose a novel concept related to the extracranial vertebral artery, addressing the unique hemodynamic pattern of the vertebrobasilar system, and present bypasses involving the extracranial V1-3 segments of the vertebral artery. We previously reviewed our modern cohort on bypass for intracranial aneurysm and showed good clinical outcome, high safety profile and excellent aneurysm obliteration rate after aneurysm treatment (8).

History indicates that improved outcomes in cerebral bypass surgery have and will continue to be achieved through practice, innovations, and technical advancements. Developments such as scissors-attached micro-forceps, presented by Yomo et al., and intraoperative infrared thermography, described by Lin et al. as an addendum to ICG-VA for evaluating bypass patency, showcase the ongoing evolution of surgical techniques. This contrast-independent method might eventually replace ICG-VA once flow distribution and quantitative analysis become available. Meanwhile, the switch from intravenous to intraarterial injection of ICG into the STA main stem, as proposed by Ni et al., allows for detecting the clear direction of blood flow and may even predict cerebral hyperperfusion syndrome.

Looking ahead, the future of bypass surgery remains exciting, particularly in treating Moyamoya vasculopathy, complex aneurysms, some skull base tumors where vessel sacrifice is necessary and well-selected cases of intracranial steno-occlusive disease. However, its technical demands necessitate personal dedication, extensive training, a substantial minimum case volume, and an interdisciplinary team for optimal outcomes (9). Ongoing research, as highlighted in the current issue of Frontiers Research Topics, plays a pivotal role in the continuous improvement of bypass surgery, ensuring that patients will continue to benefit from this important therapeutic option in the future.

## Author contributions

AK: Writing—original draft. DG: Writing—review & editing. AD: Writing—original draft.
